# Axonal degeneration and amyloid pathology predict cognitive decline beyond cortical atrophy

**DOI:** 10.1186/s13195-022-01081-w

**Published:** 2022-10-04

**Authors:** Anna Linnéa Svenningsson, Erik Stomrud, Sebastian Palmqvist, Oskar Hansson, Rik Ossenkoppele

**Affiliations:** 1 Clinical Memory Research Unit, Department of Clinical Sciences Malmö, Lund University, SE 205 02 Malmö, Sweden; 2 Memory Clinic, Skåne University Hospital, Malmö, Sweden; 3 Alzheimer Center Amsterdam, Department of Neurology, Amsterdam University Medical Center, Amsterdam Neuroscience, Amsterdam, Netherlands

**Keywords:** Resilience, Cognition, Amyloid, Neurodegeneration, Aging

## Abstract

**Background:**

Cortical atrophy is associated with cognitive decline, but the association is not perfect. We aimed to identify factors explaining the discrepancy between the degree of cortical atrophy and cognitive decline in cognitively unimpaired elderly.

**Methods:**

The discrepancy between atrophy and cognitive decline was measured using the residuals from a linear regression analysis between change in whole brain cortical thickness over time and change in a cognitive composite measure over time in 395 cognitively unimpaired participants from the Swedish BioFINDER study. We tested for bivariate associations of this residual measure with demographic, imaging, and fluid biomarker variables using Pearson correlations and independent-samples *t*-tests, and for multivariate associations using linear regression models. Mediation analyses were performed to explore possible paths between the included variables.

**Results:**

In bivariate analyses, older age (*r* = −0.11, *p* = 0.029), male sex (*t* = −3.00, *p* = 0.003), larger intracranial volume (*r* = −0.17, *p* < 0.001), carrying an *APOE*e4 allele (*t* = −2.71, *p* = 0.007), larger white matter lesion volume (*r* = −0.16, *p* = 0.002), lower cerebrospinal fluid (CSF) β-amyloid (Aβ) 42/40 ratio (*t* = −4.05, *p* < 0.001), and higher CSF levels of phosphorylated tau (p-tau) 181 (*r* = −0.22, *p* < 0.001), glial fibrillary acidic protein (GFAP; *r* = −0.15, *p* = 0.003), and neurofilament light (NfL; *r* = −0.34, *p* < 0.001) were negatively associated with the residual measure, i.e., associated with worse than expected cognitive trajectory given the level of atrophy. In a multivariate analysis, only lower CSF Aβ42/40 ratio and higher CSF NfL levels explained cognition beyond brain atrophy. Mediation analyses showed that associations between the residual measure and *APOE*e4 allele, CSF Aβ42/40 ratio, and CSF GFAP and p-tau181 levels were mediated by levels of CSF NfL, as were the associations with the residual measure for age, sex, and WML volume.

**Conclusions:**

Our results suggest that axonal degeneration and amyloid pathology independently affect the rate of cognitive decline beyond the degree of cortical atrophy. Furthermore, axonal degeneration mediated the negative effects of old age, male sex, and white matter lesions, and in part also amyloid and tau pathology, on cognition over time when accounting for cortical atrophy.

**Supplementary Information:**

The online version contains supplementary material available at 10.1186/s13195-022-01081-w.

## Background

Grey matter atrophy is associated with cognitive decline in chronological ageing [1], mild cognitive impairment (MCI) [2], and Alzheimer’s disease (AD) dementia [3, 4]. However, the association between brain structure and cognitive performance is not perfect, and there are great interindividual differences [5]. This discrepancy between pathological markers and clinical outcome has been described within the cognitive resilience and reserve framework but can also be discussed from the other perspective, by looking at risk factors for cognitive decline beyond what is explained by a specific pathological marker.

Cognitive resilience is an umbrella term that refers to multiple processes involved in avoiding (brain maintenance) and withstanding or coping with (brain and cognitive reserve) the effects of brain pathology and ageing [6]. Intracranial volume (ICV) is a proxy measure that has been used to account for brain reserve [6, 7], while education, intelligence quotient (IQ), and occupational complexity are often used as proxies for cognitive reserve [8–11]. Another method for studying reserve is the residual approach [6, 12, 13], where reserve is defined as the variance in cognition that cannot be explained by a defined pathological marker. This results in a direct, quantitative, and participant-specific measure of reserve.

As mentioned above, the opposite of resilience factors are factors that worsen cognitive decline beyond a specified pathological process. Both β-amyloid (Aβ) [14, 15] and tau [3, 16] pathology have been shown to be associated with cognitive decline, independent of grey matter atrophy. Cerebrovascular small vessel pathology is also associated with cognitive decline, although not consistently when accounting for brain volume loss [17, 18]. Cerebrospinal fluid (CSF) levels of neurofilament light (NfL), a marker of axonal integrity [19], correlate with cognitive performance [20, 21], but the interplay between NfL levels, grey matter atrophy, and cognitive performance is unclear. Studies have also shown associations between cognitive performance and CSF markers of astrocytic activation [22], microglial activation [23], and synaptic dysfunction [24], but few studies have investigated whether this is independent of cortical atrophy. Also, cardio- and cerebrovascular co-morbidities, such as diabetes mellitus [25], congestive heart failure [26], and stroke [27, 28], increase the risk of cognitive decline and dementia.

Prevention and development of non-pharmaceutical interventions to promote healthy brain ageing are of great importance, as delaying the onset of dementia or slowing cognitive decline improves quality of life and reduces public health costs [29, 30]. Through studies of resilience and risk factors, novel ways to prevent or postpone cognitive decline in the presence of pathology and atrophy can be discovered. Factors that impact future cognitive decline beyond brain atrophy are to a great extent unclear, as there have been few longitudinal studies investigating this. In this study, we calculated the discrepancy between whole brain cortical atrophy and change in global cognition at an individual level and then investigated whether resilience and risk factors, such as educational attainment, cardiovascular co-morbidities, and imaging and fluid biomarkers of amyloid, tau, and cerebrovascular pathology, astrocytic and microglial activation, and axonal and synaptic integrity, may explain this discrepancy. The residual approach renders a quantitative measure of reserve, allowing us to perform subsequent analyses to investigate possible paths between the included variables using mediation analysis.

## Methods

### Participants

Longitudinal data from cognitively unimpaired (CU) elderly participants from the Swedish BioFINDER study (https://biofinder.se; NCT01208675) were obtained. Participants performing within the normal range on cognitive testing and who did not fulfil criteria for MCI or dementia were included. This included both cognitively unimpaired participants, recruited as volunteers without subjective cognitive complaints, and people referred to the memory clinic at Skåne University Hospital or Ängelholm hospital in Sweden due to cognitive symptoms experienced by the patient and/or informant but performing within the normal range on cognitive testing (hereafter referred to as subjective cognitive decline (SCD)). The participants underwent cognitive testing, magnetic resonance imaging (MRI), and lumbar puncture every 2 years. To be included in the current study, participants had to have completed at least two MRI scans and two visits with cognitive testing, from which change in cortical thickness and change in cognitive performance were calculated. Information on demographics (age, sex, years of education), *APOE*e4 status, cardiovascular co-morbidities, CSF biomarker data, and white matter lesion (WML) volume was collected at baseline. Information on cardiovascular co-morbidities was obtained from patient history and included presence of hypertension, hyperlipidaemia, diabetes mellitus, ischemic heart disease, atrial fibrillation, congestive heart failure, and stroke/transient ischemic attack (TIA). The study was approved by the ethics committee at Lund University and the participants gave their written informed consent.

### Cognition

Since the population included in the study was cognitively unimpaired, we used a modification of the Preclinical Alzheimer’s Cognitive Composite 5 (mPACC5), which has been shown to be sensitive for detecting early cognitive changes [31], to measure cognitive performance over time. The mPACC5 was calculated using the Mini-Mental State Examination (MMSE) [32], Alzheimer’s Disease Assessment Scale – Cognitive Subscale (ADAS-Cog) delayed word recall [33], Trailmaking Test B (TMTB) [34], and Animal fluency test [35]. *Z* scores were calculated for each cognitive test, using the means and standard deviations from the same tests in a separate cohort of cognitively unimpaired participants from the BioFINDER 2 study (NCT03174938; *n* = 128). When appropriate, the results of the tests were multiplied by −1 so that higher *z* score always represented better cognition. The means from the *z* scores formed the mPACC5. As in previous publications [36], ADAS-Cog delayed word recall was weighted double since the original PACC5 includes two memory tests.

### Imaging biomarkers

The participants underwent 3-T MRI scans. Cortical reconstruction and volumetric segmentation were performed with the FreeSurfer image analysis suite, which is documented and freely available for download online (http://surfer.nmr.mgh.harvard.edu/). To extract reliable volume and thickness estimates, images were automatically processed with a longitudinal pipeline [37] in FreeSurfer. We performed manual quality assessment on the images. We computed a mean whole brain cortical thickness measure by averaging all cortical regions of interest (*n* = 34) from the Desikan-Killiany atlas [38]. Regions from both hemispheres were included and adjusted for their respective surface area. WML volume, seen as hyperintensities in T2-weighted scans, was measured using the Lesion Segmentation Tool [39, 40].

### Fluid biomarkers

Lumbar CSF samples were collected and stored in −80°C pending analyses. Levels of Aβ42, Aβ40, and phosphorylated tau 181 (p-tau181) were measured using Elecsys immunoassays [41]. The Aβ42/40 ratio was used as a measure of brain amyloid deposition [42]. Levels of NfL, glial fibrillary acidic protein (GFAP; a marker of astrocytic activation [43]), soluble triggering receptor expressed on myeloid cells 2 (sTREM2; a marker of microglial activation [44]), and neurogranin (a marker of synaptic dysfunction [45]) were measured using the NeuroToolKit panel of automated immunoassays [46].

### Statistics

We used a two-step approach, where we first calculated individual slopes for change in cortical thickness and change in cognitive performance by performing linear regressions between time (independent variable) and mean whole brain cortical thickness or mPACC5 (dependent variables), including all available data points for cortical thickness and mPACC5. Second, we performed a linear regression with change in cortical thickness as independent variable and change in mPACC5 as dependent variable, from which the standardized residuals were used as a measure of the discrepancy between brain atrophy and cognitive change over time at an individual level. A positive residual reflects a more favourable cognitive trajectory than expected given the level of atrophy, and a negative residual reflects a worse cognitive trajectory than expected. The residuals were used as the dependent variable in further analyses with baseline demographics as well as CSF and MRI biomarkers as predictors.

In bivariate analyses, Pearson correlations (*r*_p_) and independent-samples *t*-tests were performed for continuous and dichotomous variables, respectively. To look for differing results for participants with only two follow-up visits compared to those with three or more, the bivariate analyses were also performed separately for these groups. To assess independent associations between the residual measure and these variables, the variables significantly associated with the residual measure in the bivariate analyses were entered in a multivariate linear regression model adjusted for presence/absence of SCD, baseline mean whole brain cortical thickness, and baseline mPACC5. The multivariate analysis was also performed with the individual specific intercepts from the initial linear regression models for mean whole brain cortical thickness and mPACC5 as baseline measures of these variables, to account for the fact that the slopes are estimated together with an estimated baseline (intercept). We additionally tested for interaction effects between demographic variables and biomarkers on the residual measure.

To elucidate why a variable was significantly associated with the residual measure in the bivariate but not in the multivariate analysis, mediation analyses were performed using the PROCESS macro [47] in the Statistical Package for Social Sciences (SPSS), with models including either a single or multiple mediator variables. In short, this macro performs sequential linear regression analyses to establish mediation effects and then uses bootstrapping (5000 bootstrap samples) to calculate a 95% confidence interval (CI) for the direct and indirect effects of the mediation models. Effects where the bootstrapped 95% CI did not include 0 were considered significant. The designs of the mediation models were outlined in two steps. First, we checked for bivariate associations between all variables in the multivariate analysis. Second, for each variable that was significantly associated with the residual measure in the bivariate but not the multivariate analysis, a directed acyclic graph (DAG, a graphical way to display the assumptions about the relationship between variables) was drawn, incorporating the variables that were associated in the bivariate analysis. The directions of the associations were inferred from previous literature. The hypothesized mediation models (visualized as DAGs) were then tested statistically using the PROCESS macro in SPSS.

Since the Aβ42/40 ratio shows a bimodal distribution, this was used as a dichotomous variable based on a previously established cut-off level [48]. This cut-off was defined using mixture modelling, a method that has been successfully used to identify cut-offs for biomarkers of amyloid pathology [49, 50]. The distributions for WML volume as well as CSF levels of p-tau181, NfL, neurogranin, sTREM2, and GFAP were positively skewed, and the values for these variables were therefore log_10_-transformed to meet model assumptions of normal distribution. We corrected the bivariate models for multiple comparisons using false discovery rate (FDR). Statistical significance was set at *α* < 0.05. Statistical analyses were performed using SPSS Statistics for Mac (version 27).

## Results

### Sample characteristics

The characteristics of the 395 study participants are described in Table 1. The mean age was 72.4 (standard deviation (SD) 5.3) years at baseline, 59% were women, and the mean education level was 12.4 (SD 3.5) years. The median number of MRI scans was three scans and median number of cognitive test visits was three visits. Spaghetti plots showing individual slopes for whole brain cortical thickness and mPACC5 are shown in Supplementary Fig. 1.

**Table 1:** Sample characteristics for the whole sample and divided by negative or positive residual measure (better or worse cognitive trajectory than expected given the level of atrophy). Mean (standard deviation (SD)) are presented if not otherwise specified

	Whole sample (*n* = 395)	Negative residual measure (*n* = 160)	Positive residual measure (*n* = 235)
Age at baseline (years)	72.4 (5.3)	73.2 (5.6)	71.8 (5.1)
Sex (% women)	59%	49%	66%
Years of education	12.4 (3.5)	12.3 (3.6)	12.5 (3.5)
Number of MRI scans (median (range))	3 (2–5)	3 (2–4)	3 (2–5)
Mean whole brain cortical thickness at baseline (mm)	2.31 (0.11)	2.29 (0.11)	2.33 (0.10)
Change in mean whole brain cortical thickness (mm/year)	−0.012 (0.013)	−0.011 (0.015)	−0.012 (0.011)
Number of cognitive test visits (median (range))	3 (2–4)	3 (2–4)	3 (2–4)
MMSE score at baseline	28.9 (1.2)	28.8 (1.3)	28.9 (1.1)
ADAS-Cog delayed recall at baseline (incorrect answers)	2.4 (2.0)	2.8 (2.3)	2.1 (1.8)
TMTB at baseline (seconds)	99.1 (41.1)	107 (45.0)	93.9 (37.4)
Animal fluency test at baseline (correct answers)	21.2 (5.9)	20.4 (6.7)	21.8 (5.2)
mPACC5 at baseline (*z* score)	0.11 (0.71)	−0.06 (0.84)	0.22 (0.59)
Change in mPACC5 (*z* score/year)	−0.087 (0.20)	−0.25 (0.21)	0.02 (0.09)
ICV (dm^3^)	1.10 (0.13)	1.11 (0.13)	1.08 (0.12)
WML volume (cm^3^; median (range); 11 missing)	5.1 (0.01–89.5)	7.0 (0.06–89.5)	4.1 (0.01–71.5)
APOEe4 allele (% carriers; 5 missing)	33%	37%	30%
CSF Aβ42/40 (% abnormal; 1 missing)	28%	38%	22%
CSF P-tau181 (median (range); pg/ml)	18.1 (8.0–72.5)	19.5 (8.0–56.4)	17.5 (8.0–72.5)
CSF NfL (median (range); pg/ml; 9 missing)	125 (41.5–861)	142 (60.9–861)	119 (41.5–463)
CSF sTREM2 (median (range); ng/ml; 9 missing)	9.7 (3.9–21.3)	9.9 (4.6–21.0)	9.5 (3.9–21.3)
CSF neurogranin (median (range); pg/ml; 9 missing)	711 (217–2179)	728 (225–1932)	708 (217–2179)
CSF GFAP (median (range); ng/ml; 9 missing)	11.9 (3.8–44.9)	12.6 (5.1–44.9)	11.5 (3.8–35.9)
Smoking (% current or former; 56 missing)	48%	52%	45%
Hypertension (%; 1 missing)	37%	33%	39%
Hyperlipidaemia (%; 1 missing)	33%	33%	33%
Diabetes mellitus (%; 1 missing)	8.4%	8.8%	8.1%
Ischemic heart disease (&; 1 missing)	7.9%	9.4%	6.8%
Atrial fibrillation (%; 1 missing)	1.8%	1.9%	1.7%
Congestive heart failure (%; 1 missing)	1.3%	1.3%	1.3%
Stroke/TIA (%; 1 missing)	4.8%	6.3%	3.8%

*Abbreviations*: *MMSE* Mini Mental State Examination, *ADAS-Cog* Alzheimer’s Disease Assessment Scale – Cognitive subscale, *TMTB* Trailmaking Test B, *mPACC5* modified Preclinical Alzheimer’s Cognitive Composite 5, *ICV* intracranial volume, *WML* white matter lesion, *CSF* cerebrospinal fluid, *Aβ* β-amyloid, *P-tau* phosphorylated tau, *NfL* neurofilament light, *sTREM2* soluble triggering receptor expressed on myeloid cells 2, *GFAP* glial fibrillary acidic protein, *TIA* transient ischemic attack

### Residual measure

Across the whole group, the mean change in mean whole brain cortical thickness was −0.012 (SD 0.013) mm/year and mean change in mPACC5 was −0.087 (SD 0.20) *z* scores/year. Changes in these measures were modestly correlated (*r*_p_ = 0.3, *p* < 0.001; Supplementary Fig. 2).

### Bivariate analyses

In bivariate analyses, older age (*r* = −0.11, *p* = 0.029), male sex (*t* = −3.00, *p* = 0.003), larger ICV (*r* = −0.17, *p* < 0.001), larger WML volume (*r* = −0.16, *p* = 0.002), carrying an *APOE*e4 allele (*t* = −2.71, *p* = 0.007), having a lower Aβ42/40 ratio (*t* = −4.05, *p* < 0.001), and higher levels of p-tau181 (*r* = −0.22, *p* < 0.001), GFAP (*r* = −0.15, *p* = 0.003), and NfL (*r* = −0.34, *p* < 0.001) were significantly associated with the residual measure (i.e. worse than expected cognitive trajectory given the level of atrophy). Only age did not survive correction for multiple comparisons. Years of education (*r* = 0.04, *p* = 0.431), smoking (*t* = −0.05, *p* = 0.958), cardiovascular co-morbidities (*t* = −1.4 to 1.08, *p* = 0.178–0.903), and CSF levels of sTREM2 (*r* = −0.07, *p* = 0.183) and neurogranin (*r* = −0.10, *p* = 0.059) were not associated with the residual measure (Table 2). For most variables, the general directions of the associations were the same for participants with two MRI scans compared to those with three or more, as well as for participants with two cognitive test visits compared to those with three or more. The direction of association differed between the groups for smoking, diabetes mellitus, ischaemic heart disease, and stroke/TIA, but none of these results were statistically significant (Supplementary Tables 1A and B).

**Table 2:** Bivariate analyses. Associations between the residual measure and demographic, co-morbidity, and biomarker variables. Pearson correlation coefficients with bootstrapped 95% CIs for continuous variables and independent samples *t* values for binary variables with *p* values before and after FDR correction are presented

	Pearson correlation coefficient (95% CI)	*T* value	*P* value	*P* value after FDR correction
**Age at baseline**	**−0.11 (−0.21 to −0.02)**	-	**0.029**	0.064
**Sex**	-	**−3.00**	**0.003**	**0.009**
Years of education	0.04 (**−**0.06 to 0.14)	-	0.431	0.571
**ICV**	**−0.17 (−0.27 to −0.07)**	-	**< 0.001**	**0.005**
**WML volume**	**−0.16 (−0.25 to −0.07)**	-	**0.002**	**0.008**
**APOEe4 allele**	-	**−2.71**	**0.007**	**0.018**
**Low CSF Aβ42/40 ratio**	-	**−4.05**	**< 0.001**	**< 0.001**
**CSF P-tau181**	**−0.22 (−0.32 to −0.12)**	-	**< 0.001**	**< 0.001**
**CSF NfL**	**−0.34 (−0.44 to −0.23)**	-	**< 0.001**	**< 0.001**
CSF sTREM2	**−**0.07 (**−**0.16 to 0.02)	-	0.183	0.305
CSF neurogranin	**−**0.10 (**−**0.19 to 0.00)	-	0.059	0.118
**CSF GFAP**	**−0.15 (-0.26 to −0.05)**	-	**0.003**	**0.009**
Smoking (current or former)	-	**−**0.05	0.958	0.958
Hypertension	-	1.08	0.281	0.432
Hyperlipidaemia	-	0.88	0.382	0.546
Diabetes mellitus	-	**−**0.35	0.726	0.807
Ischemic heart disease	-	**−**0.12	0.903	0.951
Atrial fibrillation	-	**−**0.74	0.457	0.571
Congestive heart failure	-	0.56	0.575	0.676
Stroke/TIA	-	**−**1.40	0.178	0.305

*Abbreviations*: *CI* confidence interval, *FDR* false discovery rate, *ICV* intracranial volume, *WML* white matter lesion, *Aβ* β-amyloid, *P-tau* phosphorylated tau, *NfL* neurofilament light, *sTREM2* soluble triggering receptor expressed on myeloid cells 2, *GFAP* glial fibrillary acidic protein, *TIA* transient ischemic attack

### Multivariate analysis

**Fig. 1:**
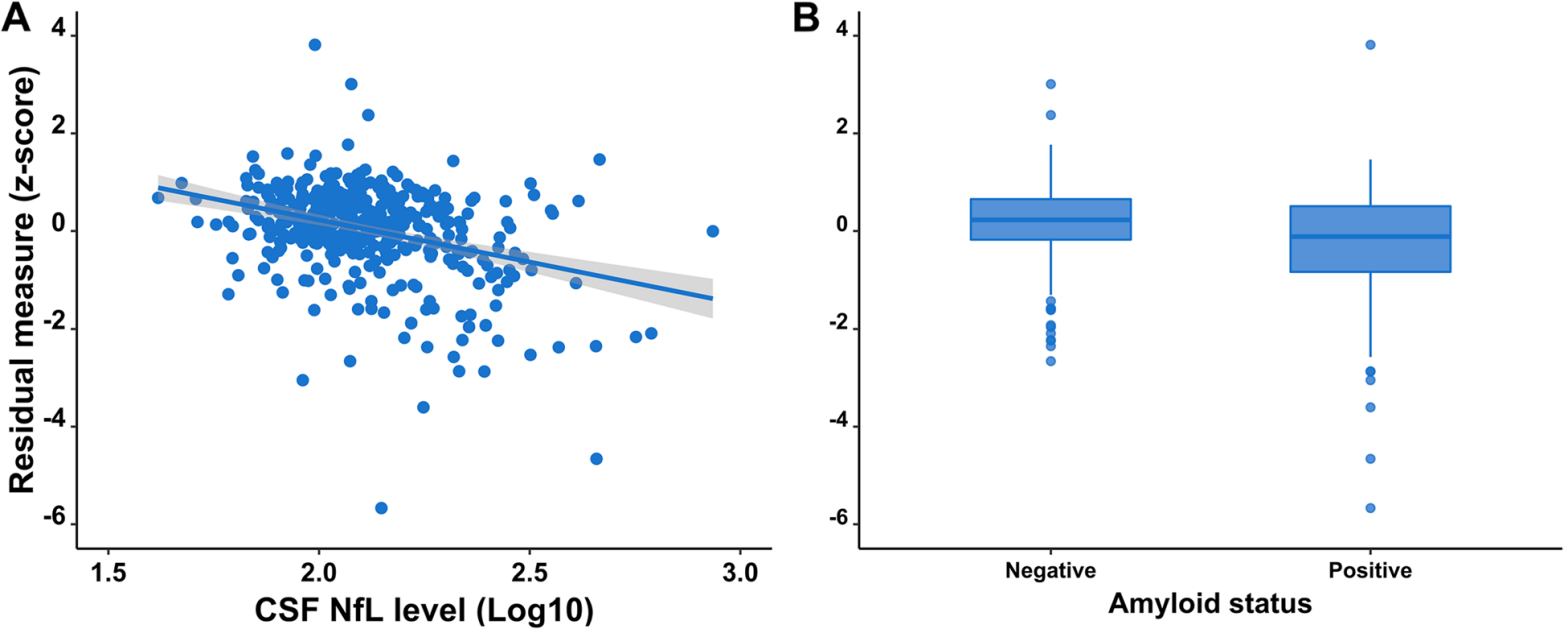
Scatterplot showing the negative association between baseline CSF levels of NfL (log_10_-transformed) and the residual measure (**A**), and boxplot showing that participants with normal Aβ42/40 ratio have higher residual measure than participants with abnormal Aβ42/40 ratio at baseline (**B**). Abbreviations: CSF, cerebrospinal fluid; NfL, neurofilament light; Aβ, β-amyloid

A multivariate linear regression analysis was performed, including the residual measure as dependent variable and the variables significantly associated with the residual measure in bivariate analyses (see above) as independent variables, controlling for presence/absence of SCD, baseline mean whole brain cortical thickness, and baseline mPACC5. In this analysis, only higher NfL levels (*β* = −0.20 (95% CI −0.34 to (−0.05)), *p* = 0.009) and having a lower Aβ42/40 ratio (*β* = −0.11 (95% CI −0.23 to (−0.004)), *p* = 0.049) were associated with the residual measure (Table 3, Fig. 1A, B). The results were similar when using the individual specific intercepts for whole brain cortical thickness and mPACC5 from the initial linear regression models as baseline measures of these variables (Supplementary Table 2).

**Table 3:** Multivariate analysis. Multivariate linear regression model with the residual measure as dependent variable and the variables statistically significant in bivariate analyses (Table 2) as independent variables, controlling for presence/absence of subjective cognitive decline, baseline mean whole brain cortical thickness, and baseline mPACC5. Standardized beta coefficients with bootstrapped 95% CIs are presented

	Standardized beta coefficient (95% CI)	*P* value
Age at baseline	0.02 (−0.10 to 0.16)	0.768
Male sex	0.01 (−0.13 to 0.11)	0.813
ICV	−0.10 (−0.21 to 0.03)	0.108
WML volume	0.003 (−0.10 to 0.11)	0.960
APOEe4 allele	−0.04 (−0.15 to 0.06)	0.405
**Abnormal CSF Aβ42/40**	−**0.11 (**−**0.23 to **−**0.004)**	**0.049**
CSF P-tau181	−0.03 (−0.19 to 0.12)	0.687
**CSF NfL**	−**0.20 (**−**0.34 to **−**0.05)**	**0.009**
CSF GFAP	−0.01 (−0.11 to 0.12)	0.920

*Abbreviations*: *CI* confidence interval, *ICV* intracranial volume, *WML* white matter lesion, *Aβ* β-amyloid, *P-tau* phosphorylated tau, *NfL* neurofilament light, *GFAP* glial fibrillary acidic protein, *mPACC5* modified Preclinical Alzheimer’s Cognitive Composite 5

There were no significant two-way interactions for ICV, WML volume, *APOE*e4, Aβ42/40 ratio, or levels of p-tau181, GFAP, or NfL with age, sex, or education on the residual measure (Supplementary Table 3).

### Mediation analyses

As a first step, we constructed the directed acyclic graphs (DAGs) for variables that were significantly associated with the residual measure in bivariate but not multivariate analysis to visualize hypothesized paths of mediation (Supplementary Fig. 3). These were based on associations in this sample between the variables significant in bivariate analyses (Supplementary Table 4) and the hypothesized directions of the associations were based on either the inherent nature of the variables (i.e. if there is a temporal association between age or sex with any of the biomarkers, age or sex would come first in the ordering) or hypotheses based on existing literature (i.e. the amyloid cascade hypothesis [51], GFAP being closely linked to amyloid pathology [52], and WMLs preceding brain atrophy [53]). ICV could not, based on previous literature, be fitted in a hypothesized causal model predicting NfL and was therefore included as a covariate instead.

 As a second step, we statistically tested the hypothesized mediation models using the PROCESS macro in SPSS. This showed that the associations between the residual measure and *APOE*e4 allele, lower Aβ42/40 ratio (partially), GFAP levels, and p-tau181 levels were mediated by levels of NfL, as were the associations with the residual measure for age, sex, and WML volume. More detailed results from these analyses are shown in Table 4.

**Table 4:** Mediation analyses testing hypothesized paths (Supplementary Fig. 3). Variables significantly associated with the residual measure in bivariate analyses (Table 2) that were not included as mediators were included as covariates as were the presence/absence of subjective cognitive decline, baseline mean whole brain cortical thickness, and baseline mPACC5. Significant mediation paths (defined as bootstrapped 95% CI not including 0) are highlighted in bold

	Included potential mediators	Path	Beta (95% CI)	Standardized beta (95% CI)^a^
Age	p-tau181, WML volume, NfL			
Direct effect			0.003 (-0.018 to 0.026)	
Total indirect effects			**-0.010 (-0.022 to -0.0002)**	**-0.058 (-0.127 to -0.001)**
Indirect effects		Age – P-tau181 – NfL – residual	-0.0006 (-0.002 to 0.0004)	-0.003 (-0.014 to 0.002)
		Age – NfL – residual	**-0.007 (-0.016 to -0.001)**	**-0.043 (-0.094 to -0.009)**
		Age – WML volume – NfL – residual	**-0.002 (-0.004 to -0.0003)**	**-0.012 (-0.025 to -0.002)**
Male sex	WML volume, NfL			
Direct effect			0.029 (-0.209 to 0.267)	
Total indirect effect			-0.045 (-0.112 to 0.006)	-0.048 (-0.120 to 0.006)
Indirect effects		Sex – NfL – residual	**-0.050 (-0.116 to -0.006)**	**-0.054 (-0.125 to -0.007)**
		Sex – WML volume – NfL – residual	0.006 (-0.001 to 0.017)	0.006 (-0.002 to 0.018)
WML volume	NfL			
Direct effect			0.004 (-0.172 to 0.181)	
Total indirect effect			**-0.049 (-0.101 to -0.009)**	**-0.034 (-0.070 to -0.006)**
Indirect effects		WML volume – NfL – residual	**-0.049 (-0.101 to -0.009)**	**-0.034 (-0.070 to -0.006)**
APOEe4 allele	Aβ42/40^b^, GFAP, P-tau181, NfL			
Direct effect			-0.067 (-0.285 to 0.152)	
Total indirect effect			**-0.173 (-0.315 to -0.051)**	**-0.184 (-0.324 to -0.057)**
Indirect effects		APOE4 – Aβ42/40 – GFAP – P-tau181 – NfL – residual	**-0.0038 (-0.0087 to -0.0005)**	**-0.0041 (-0.010 to -0.0006)**
		APOE4 – Aβ42/40 – GFAP – NfL – residual	**-0.0069 (-0.0164 to -0.001)**	**-0.0074-(-0.0174 to -0.0011)**
		APOE4 – Aβ42/40 – P-tau181 – NfL – residual	**-0.0237 (-0.050 to -0.006)**	**-0.025 (-0.054 to -0.006)**
		APOE4 – Aβ42/40 – NfL – residual	0.004 (-0.016 to 0.034)	0.004 (-0.018 to 0.037)
		APOE4 – Aβ42/40 – residual	**-0.129 (-0.315 to -0.051)**	**-0.137 (-0.268 to -0.021)**

*Abbreviations*: *WML* white matter lesion, *Aβ* β-amyloid, *P-tau* phosphorylated tau, *GFAP* glial fibrillary acidic protein, *NfL* neurofilament light, *mPACC5* modified Preclinical Alzheimer’s Cognitive Composite 5, *CI* confidence interval

^a^For dichotomous independent variables (sex, APOEe4 allele, and pathological Aβ42/40), these are partially standardized beta coefficients (original metric for *x* but standardized *y*)

^b^Since the macro does not allow dichotomous mediators, continuous Aβ42/40 ratio was used for this mediation analysis

## Discussion

Using a residual approach to quantify the discrepancy between whole brain cortical atrophy (change in cortical thickness) and change in cognitive performance in a sample of cognitively unimpaired elderly, we show that this discrepancy is partly explained by levels of NfL and lower Aβ42/40 ratio in CSF. Also, levels of NfL mediated associations between the residual measure and age, sex, WML volume, *APOE*e4 allele, Aβ42/40 ratio, and levels of GFAP and p-tau181. Altogether, our results suggest that axonal degeneration and amyloid pathology affect the rate of cognitive decline beyond the degree of cortical atrophy.

**Fig. 2:**
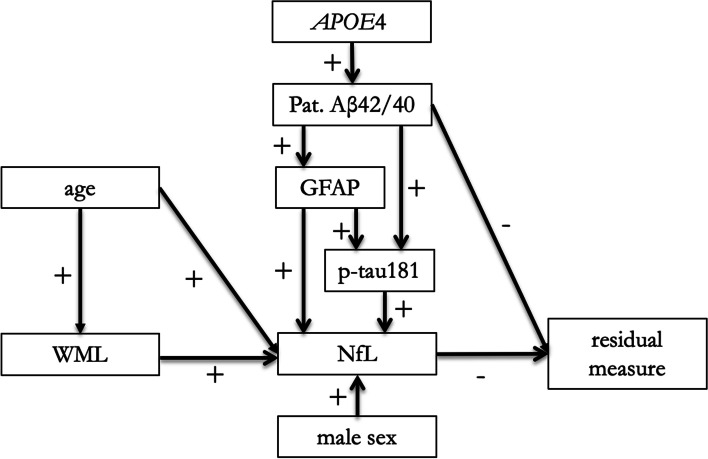
Aggregated, hypothetical model of the associations between different predictors and the residual measure based on the results from the mediation analyses (Table 4). Plus indicates a positive association and minus a negative association between the variables. Abbreviations: WML, white matter lesion; Aβ, β-amyloid; GFAP, glial fibrillary acidic protein; P-tau, phosphorylated tau; NfL, neurofilament light

NfL levels in CSF correlate with cognitive performance [20, 21] and NfL is often considered a possible marker of neurodegenerative processes (akin to brain atrophy on MRI) [54–56]. Here we show that NfL and cortical atrophy provide complementary information when predicting cognitive change, suggesting that they reflect overlapping yet distinct processes in the brain. Levels of NfL also mediated the associations between the residual measure and several other predictors (age, sex, WML volume, and AD and astrocytic biomarkers), which could be expected given that NfL is a sensitive, but non-specific marker of neurological disorders [57], is associated with older age and male sex in CU [58, 59], and is closely associated with global cognitive performance and future cognitive decline [20, 60]. In Fig. 2, we present an aggregated, hypothetical model including the significant mediation effects of NfL from Table 4. It is, however, important to emphasize that the possible associations in this model have not been tested statistically as a combined model, and the model is to be seen as a hypothetical model to be tested in further studies.

The presence of AD pathology was another predictor of worse than expected cognitive decline given the degree of brain atrophy, as presence of an *APOE*e4 allele, as well as amyloid and tau pathology were associated with the residual measure in bivariate analyses, and amyloid pathology also in multivariate analysis. This atrophy independent association between amyloid pathology and the residual measure indicate amyloid-related effects on cognition that are independent of gross cortical atrophy or axonal degeneration. Instead, amyloid pathology could induce network disruption (i.e. functional rather than structural) alterations [61]. Mediation analyses showed that the association between the residual measure and CSF p-tau181 was mediated by CSF levels of NfL, and both p-tau181 and NfL levels partially mediated the association between amyloid pathology and the residual measure. This is in line with existing evidence of especially tau pathology being associated with neurodegeneration [15, 62, 63], which also could also explain why CSF p-tau181 did not remain significant in the multivariate model, i.e. when taking atrophy into account, as tau pathology did not explain additional cognitive decline beyond atrophy.

Older age was associated with a worse than expected cognitive trajectory given the level of cortical atrophy in univariate, but not multivariate, analyses. Considering chronological age as a proxy for many different processes that occur in the ageing brain, this observation is in line with our a priori hypotheses. With higher age, changes within and between brain networks have been observed [64, 65], which could explain the association between age and cognitive performance beyond atrophy. The association was mediated by levels of NfL, in part via cerebrovascular pathology measured by WML volume. NfL has been shown to correlate with white matter changes in elderly individuals with and without dementia [66, 67]. In contrast, a recent study showed no association between WML volume and levels of NfL, but this study included individuals at all ages [68].

In bivariate analyses, females showed more favourable cognitive trajectories than expected on a group level. Previous studies using different definitions and measures of reserve and resilience have indicated clear sex differences in cognitive resilience. For example, women showed relative preservation of cortical thickness when exposed to tau pathology compared to men [69] indicating greater brain resilience, and in a cohort of cognitively unimpaired elderly, women had higher entorhinal cortical tau than men [70], indicating greater cognitive resilience. These results indicate that women can tolerate more aggregated tau in their brains before exhibiting neurodegeneration and cognitive decline. The negative association between male sex and cognition was mediated by levels of NfL. ICV, often used as a marker of brain reserve [6, 7], was negatively associated with the residual measure in bivariate analysis. To some degree, this was due to confounding by sex with men having lower residual measure and larger ICV than women.

CSF levels of GFAP were associated with the residual measure in bivariate but not multivariate analysis. It also partially mediated the associations between amyloid pathology and subsequent p-tau181 levels, NfL levels, and the residual measure. GFAP in CSF is associated with cognitive performance [22] and is increased in different neurodegenerative diseases compared to controls [71] and in CU individuals with AD pathology compared to those without [59]. No association was seen between the residual measure and CSF levels of sTREM2 or neurogranin. For sTREM2, the association with neurodegeneration and cognition is not consistent, with studies showing both increased levels in AD patients compared to controls [72] as well as an association between higher levels and attenuated cognitive decline in individuals with AD [23]. Neurogranin is associated with cognitive decline [24], but the lack of association with cognitive trajectory in our study could be due to its high expression in associative cortical regions [73], since the association between cognition and cortical atrophy/function has already been accounted for in our analysis.

There was no significant association between the residual measure and education, which is often used as a proxy for cognitive reserve [9]. However, a recent review concluded that education level does not affect the rate of cognitive decline; instead, it showed that people with higher education start at a higher cognitive level, and therefore, they have more to lose before reaching the level of dementia [74]. The results from the present study are in line with that conclusion, with education not being associated with better or worse cognitive trajectory over time given the rate of atrophy.

No association was found for any cardiovascular comorbidity or smoking with the residual measure. Previous studies have shown associations for diabetes mellitus [25], congestive heart failure [26], and stroke [27, 28], with risk of cognitive decline and dementia, but there are few studies investigating the association when accounting for brain atrophy. The lack of association for diabetes (8.4%) and stroke/TIA (4.8%) could be due to lack of power in this sample of 395 participants, but hypertension (37%), hyperlipidaemia (33%), and smoking (48%) were frequently observed in our sample.

## Limitations

Strengths of this study include the longitudinal design and the well-characterized and relatively large sample of participants. However, there are limitations. First, measuring changes in cortical thickness and cognitive performance in parallel, when atrophy is hypothesized to precede cognitive decline, may affect the estimated association between the two measures. Second, even if the longitudinal design is of great value to assess changes over time, there is a possibility of attrition bias, with the inherent risk of selective drop-out of individuals showing the most rapid cognitive deterioration during follow-up. Third, for the mediation analysis, the main part of the variables included are measured at the same time point, even though the dependent variable is based on longitudinal data. When using cross-sectional data in mediation, the results must be interpreted with caution. This issue is addressed by building DAGs based on previous knowledge and hypotheses about causal associations between the variables, but they are built on assumptions on the directions of associations and do not guarantee that these directions are true. Fourth, the residual approach for studying cognitive resilience can be discussed. It is a useful method since it directly reflects the concept of interest, i.e. the variance in cognition not explained by a specified pathology, and renders a quantitative measure of resilience. However, one needs to bear in mind that apart from resilience, it also reflects random error, and the results are highly dependent on the variables chosen for the regression analysis and the sample participants. Therefore, results must be interpreted with caution.

## Conclusions

In conclusion we show, that in cognitively unimpaired elderly individuals, axonal degeneration and amyloid pathology predict cognitive decline beyond what can be explained by cortical atrophy. This is in line with previous findings indicating that amyloid pathology has a small but significant association with cognition independent of atrophy. It confirms that neurodegeneration is closely linked to cognition, but also suggests that different measures used to assess neurodegeneration, e.g. grey matter atrophy and axonal degeneration, provide complementary information when predicting cognitive performance. Additionally, these results suggest that axonal degeneration mediates the negative effects of old age, male sex, WML volume, astrocytic activation, tau, and in part also amyloid pathology, on cognition over time when accounting for cortical atrophy.

## Supplementary Information

**Additional file 1: Supplementary Tables 1A and B.** Bivariate analyses divided into participants with two or more than two MRI scans or cognitive visits. **Supplementary Table 2.** Multivariate analysis. **Supplementary Table 3.** Interaction effects. **Supplementary Table 4.** Associations between all variables significantly associated with the residual measure in bivariate analyses. **Supplementary Figure 1.** Spaghetti plots showing individual trajectories over time for mean whole brain cortical thickness and mPACC5. **Supplementary Figure 2.** Scatterplot showing the positive association between change in mean whole brain cortical thickness and change in global cognition. **Supplementary Figure 3.** Directed acyclic graphs (DAGs) constructed for inclusion of variables in mediation analyses.

## Data Availability

Anonymized data will be shared by request from a qualified academic investigator for the sole purpose of replicating procedures and results presented in the article and as long as data transfer is in agreement with EU legislation on the general data protection regulation and decisions by the Swedish Ethical Review Authority and Region Skåne, which should be regulated in a material transfer agreement.

## Abbreviations

AD: Alzheimer’s disease
ADAS-Cog: Alzheimer’s Disease Assessment Scale – Cognitive Subscale
APOEe4: Apolipoprotein E4
Aβ: β-Amyloid
CI: Confidence interval
CSF: Cerebrospinal fluid
CU: Cognitively unimpaired
DAG: Directed acyclic graph
FDR: False discovery rate
GFAP: Glial fibrillary acidic protein
ICV: Intracranial volume
IQ: Intelligence quotation
MCI: Mild cognitive impairment
MMSE: Mini-Mental State Examination
MRI: Magnetic resonance imaging
NfL: Neurofilament light
PACC: Preclinical Alzheimer’s Cognitive Composite
P-tau181: Tau phosphorylated at threonine 181
SCD: Subjective cognitive decline
SD: Standard deviation
SPSS: Statistical Package for Social Sciences
sTREM2: Soluble triggering receptor expressed on myeloid cells 2
TIA: Transient ischemic attack
TMTB: Trailmaking Test B
WML: White matter lesion
