# Supplementary material for: Axonal degeneration and amyloid pathology predict cognitive decline beyond cortical atrophy
Source: Alzheimers Res Ther. 2022 Oct 4;14:144. doi: 10.1186/s13195-022-01081-w (PMC9531524; doi:10.1186/s13195-022-01081-w)
Supplement: Supplementary file 1 — Additional file 1: Supplementary Tables 1A and B. Bivariate analyses divided into participants with two or more than two MRI scans or cognitive visits. Supplementary Table 2. Multivariate analysis. Supplementary Table 3. Interaction effects. Supplementary Table 4. Associations between all variables significantly associated with the residual measure in bivariate analyses. Supplementary Figure 1. Spaghetti plots showing individual trajectories over time for mean whole brain cortical thickness and mPACC5. Supplementary Figure 2. Scatterplot showing the positive association between change in mean whole brain cortical thickness and change in global cognition. Supplementary Figure 3. Directed acyclic graphs (DAGs) constructed for inclusion of variables in mediation analyses. [file 13195_2022_1081_MOESM1_ESM.docx]

**Supplementary tables**

Supplementary Table 1A

|  | Participants with 2 MRI scans (n=87) | | | | Participants with > 2 MRI scans (n=308) | | | |
| --- | --- | --- | --- | --- | --- | --- | --- | --- |
|  | Pearson correlation coefficient | T value | P value | Pearson correlation coefficient | | T value | P value |  |
| Age at baseline | -0.142 |  | 0.189 | -0.084 | |  | 0.141 |  |
| Male sex |  | **-2.6** | **0.012** |  | | **-2.0** | **0.049** |  |
| Years of education | 0.066 |  | 0.544 | 0.072 | |  | 0.207 |  |
| ICV | **-0.258** |  | **0.016** | **-0.147** | |  | **0.010** |  |
| WML volume | -0.222 |  | 0.052 | **-0.139** | |  | **0.015** |  |
| APOEε4 allele |  | -1.2 | 0.236 |  | | **-2.5** | **0.013** |  |
| Low CSF Aβ42/40 ratio |  | -1.5 | 0.135 |  | | **-3.8** | **<0.001** |  |
| CSF P-tau181 | -0.153 |  | 0.157 | **-0.245** | |  | **<0.001** |  |
| CSF NfL | **-0.365** |  | **<0.001** | **-0.322** | |  | **<0.001** |  |
| CSF sTREM2 | -0.114 |  | 0.303 | -0.046 | |  | 0.425 |  |
| CSF neurogranin | -0.045 |  | 0.684 | **-0.125** | |  | **0.030** |  |
| CSF GFAP | -0.111 |  | 0.316 | **-0.166** | |  | **0.004** |  |
| Smoking (current or former) |  | 1.3 | 0.213 |  | | -0.78 | 0.437 |  |
| Hypertension |  | 0.11 | 0.914 |  | | 1.3 | 0.181 |  |
| Hyperlipidaemia |  | 0.84 | 0.404 |  | | 0.52 | 0.605 |  |
| Diabetes mellitus |  | -0.46 | 0.646 |  | | 0.068 | 0.946 |  |
| Ischemic heart disease |  | 0.46 | 0.650 |  | | -0.46 | 0.648 |  |
| Atrial fibrillation | - | - | - |  | | -0.93 | 0.354 |  |
| Congestive heart failure |  | 0.66 | 0.513 |  | | 0.18 | 0.860 |  |
| Stroke/TIA |  | -0.51 | 0.645 |  | | -1.3 | 0.205 |  |

Supplementary Table 1B

|  | Participants with 2 cognitive test visits (n=40) | | | Participants with > 2 cognitive test visits (n=355) | | |
| --- | --- | --- | --- | --- | --- | --- |
|  | Pearson correlation coefficient | T value | P value | Pearson correlation coefficient | T value | P value |
| Age at baseline | -0.041 |  | 0.801 | **-0.122** |  | **0.022** |
| Male sex |  | **-2.3** | **0.027** |  | **-2.4** | **0.016** |
| Years of education | 0.083 |  | 0.610 | 0.031 |  | 0.565 |
| ICV | **-0.404** |  | **0.010** | **-0.145** |  | **0.006** |
| WML volume | -0.154 |  | 0.371 | **-0.162** |  | **0.002** |
| APOEε4 allele |  | -1.2 | 0.231 |  | -2.5 | 0.015 |
| Low CSF Aβ42/40 ratio |  | -1.4 | 0.173 |  | **-3.8** | **<0.001** |
| CSF P-tau181 | -0.173 |  | 0.287 | **-0.227** |  | **<0.001** |
| CSF NfL | -0.301 |  | 0.063 | **-0.346** |  | **<0.001** |
| CSF sTREM2 | -0.199 |  | 0.225 | -0.048 |  | 0.372 |
| CSF neurogranin | -0.090 |  | 0.584 | -0.096 |  | 0.075 |
| CSF GFAP | -0.275 |  | 0.090 | **-0.137** |  | **0.010** |
| Smoking (current or former) |  | 0.61 | 0.550 |  | -0.39 | 0.698 |
| Hypertension |  | 0.64 | 0.525 |  | 0.87 | 0.386 |
| Hyperlipidaemia |  | 1.0 | 0.305 |  | 0.46 | 0.647 |
| Diabetes mellitus |  | 0.13 | 0.899 |  | -0.45 | 0.650 |
| Ischemic heart disease |  | 0.60 | 0.549 |  | -0.49 | 0.623 |
| Atrial fibrillation | - | - | - |  | -0.84 | 0.404 |
| Congestive heart failure |  | 0.51 | 0.616 |  | 0.23 | 0.819 |
| Stroke/TIA |  | 0.52 | 0.604 |  | -1.6 | 0.121 |

Supplementary Tables 1A and B. Bivariate analyses. Associations between the residual measure and demographic, co-morbidity, and biomarker variables. Results are presented separately for participants with two and more than two MRI scans or cognitive visits. No participants in the “2 MRI scans” or “2 cognitive test visits” groups had atrial fibrillation. Corresponds to Table 2 in the main manuscripts.

Abbreviations: ICV – intracranial volume; WML – white matter lesion; Aβ – β-amyloid; P-tau – phosphorylated tau; NfL – neurofilament light; sTREM2 – soluble triggering receptor expressed on myeloid cells 2; GFAP – glial fibrillary acidic protein; TIA – transient ischemic attack.

Supplementary Table 2

|  | Standardized beta coefficient | P value |
| --- | --- | --- |
| Age at baseline | 0.003 | 0.962 |
| Male sex | 0.012 | 0.847 |
| ICV | -0.104 | 0.118 |
| WML volume | -0.003 | 0.967 |
| APOEε4 allele | -0.044 | 0.417 |
| **Abnormal CSF Aβ42/40** | **-0.127** | **0.044** |
| CSF P-tau181 | -0.043 | 0.541 |
| **CSF NfL** | **-0.217** | **<0.001** |
| CSF GFAP | -0.011 | 0.856 |

Supplementary Table 2. Multivariate analysis. Multivariate linear regression model with the residual measure as dependent variable and the variables statistically significant in bivariate analyses (Table 2) as independent variables, controlling for presence/absence of subjective cognitive decline, and intercept for mean whole brain cortical thickness and mPACC5. Corresponds to Table 3 from the main manuscript.

Abbreviations: ICV – intracranial volume; WML – white matter lesion; Aβ – β-amyloid; P-tau – phosphorylated tau; NfL – neurofilament light; GFAP – glial fibrillary acidic protein; mPACC5 – modified Preclinical Alzheimer´s Cognitive Composite 5.

Supplementary Table 3

|  | Age | | | Sex | | | Education | | |
| --- | --- | --- | --- | --- | --- | --- | --- | --- | --- |
|  |  | B | P value |  | B | P value |  | B | P value |
| ICV | Age*ICV | 0.012 | 0.859 | Sex*ICV | 0.717 | 0.453 | Education*ICV | -0.085 | 0.404 |
| WML volume | Age*WML volume | -0.003 | 0.822 | Sex*WML volume | 0.055 | 0.721 | Education*WML volume | 0.024 | 0.236 |
| APOEε4 | Age*APOEε4 | -0.006 | 0.775 | Sex*APOEε4 | 0.191 | 0.355 | Education*APOEε4 | -0.016 | 0.572 |
| Abnormal Aβ42/40 | Age*pathological Aβ42/40 | 0.011 | 0.584 | Sex*abnormal Aβ42/40 | -0.131 | 0.534 | Education*abnormal Aβ42/40 | -0.034 | 0.228 |
| P-tau181 | Age*P-tau181 | 0.071 | 0.163 | Sex*P-tau181 | -0.934 | 0.103 | Education*P-tau181 | -0.099 | 0.218 |
| NfL | Age*NfL | 0.011 | 0.793 | Sex*NfL | 0.824 | 0.095 | Education*NfL | -0.011 | 0.849 |
| GFAP | Age*GFAP | 0.037 | 0.470 | Sex*GFAP | -0.114 | 0.854 | Education*GFAP | 0.032 | 0.728 |

Supplementary Table 3. Results from linear regression models including the respective predictors and interaction term, controlling for presence/absence of subjective cognitive decline, baseline mean whole brain cortical thickness, and baseline mPACC5.

Abbreviations: ICV – intracranial volume; WML – white matter lesion; Aβ – β-amyloid; P-tau – phosphorylated tau; NfL – neurofilament light; GFAP – glial fibrillary acidic protein; mPACC5 – modified Preclinical Alzheimer´s Cognitive Composite 5.

Supplementary Table 4

|  | Sex | ICV | WML volume | APOEε4 | Abnormal Aβ42/40 | P-tau181 | NfL | GFAP |
| --- | --- | --- | --- | --- | --- | --- | --- | --- |
| Age | t = -0.47, p = 0.636 | r = 0.04, p = 0.447 | **r = 0.42, p < 0.001** | t = 1.51, p = 0.133 | t = -1.67, p = 0.095 | **r = 0.23, p < 0.001** | **r = 0.38, p < 0.001** | **r = 0.35, p < 0.001** |
| Sex | - | **t = 14.88, p < 0.001** | **t = 2.76, p = 0.006** | Χ^2^ = 1.45, p = 0.229 | Χ^2^ = 2.29, p = 0.130 | t = 1.45, p = 0.150 | **t = 3.73, p < 0.001** | t = 0.37, p = 0.712 |
| ICV | - | - | **r = 0.35, p < 0.001** | t = -0.94, p = 0.348 | t = -1.27, p = 0.208 | r = -0.06, p = 0.259 | **r = 0.19, p < 0.001** | **r = 0.12, p = 0.024** |
| WML volume | - | - | - | t = 1.24, p = 0.217 | t = -1.65, p = 0.099 | r = -0.04, p = 0.445 | **r = 0.32, p < 0.001** | **r = 0.23, p < 0.001** |
| APOEε4 | - | - | - | - | **Χ^2^ = 75.1, p < 0.001** | **t = -4.44, p < 0.001** | t = -1.87, p = 0.062 | t = -1.81, p = 0.071 |
| Abnormal Aβ42/40 | - | - | - | - | - | **t = -10.00, p < 0.001** | **t = -4.92, p < 0.001** | **t = -5.02, p < 0.001** |
| P-tau181 | - | - | - | - | - | - | **r = 0.48, p < 0.001** | **r = 0.49, p < 0.001** |
| NfL | - | - | - | - | - | - | - | **r = 0.48, p < 0.001** |

Supplementary Table 4. Associations between all variables significantly associated with the residual measure in bivariate analyses (Table 2). For associations between two binary variables, Chi-Square test, between one binary and one continuous variable, independent samples t test, and between two continuous variables Pearson correlation was used.

Abbreviations: ICV – intracranial volume; WML – white matter lesion; Aβ – β-amyloid; P-tau – phosphorylated tau; NfL – neurofilament light; GFAP – glial fibrillary acidic protein.

**Supplementary figures**

Supplementary Figure 1A


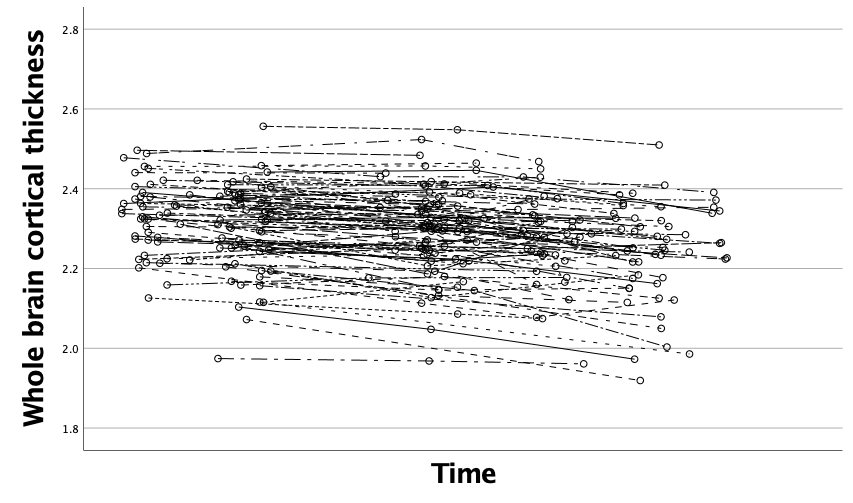


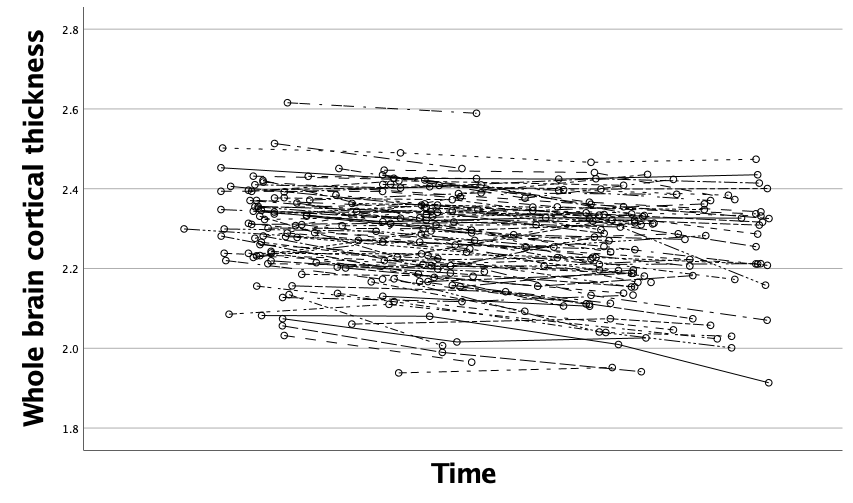

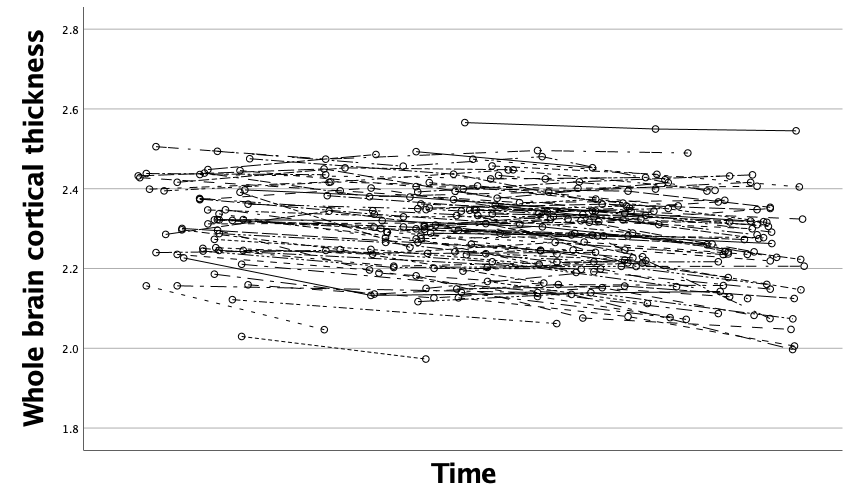

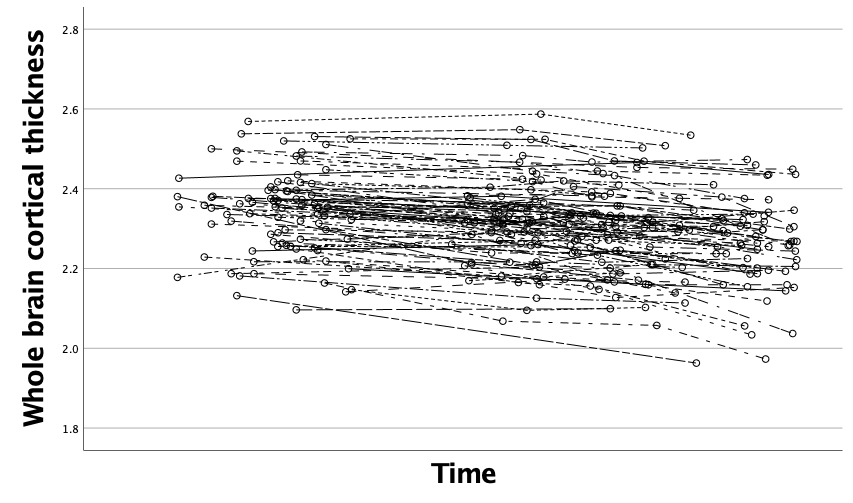


Supplementary Figure 1B


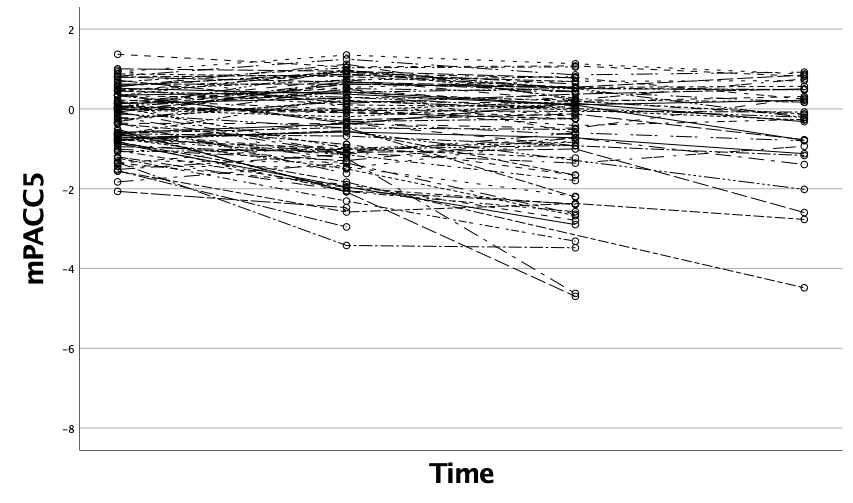

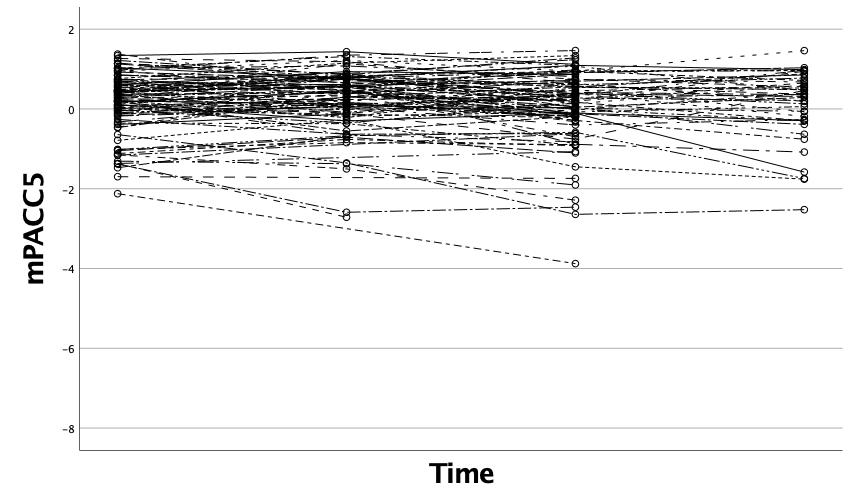


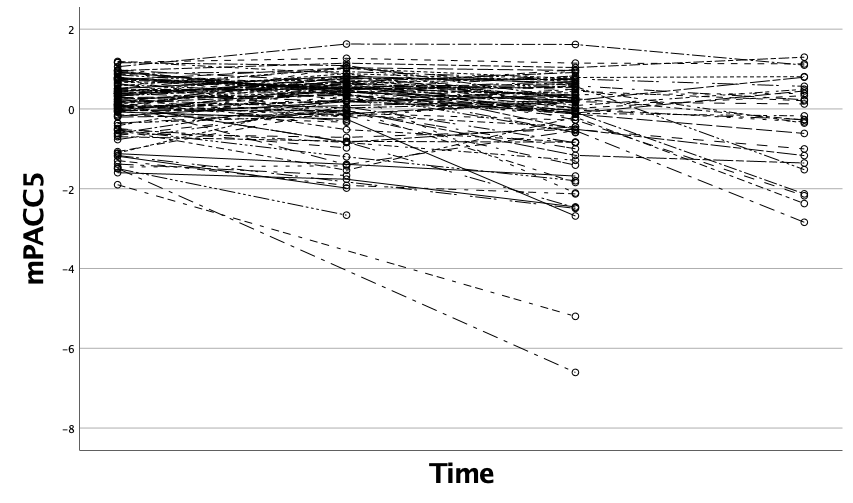

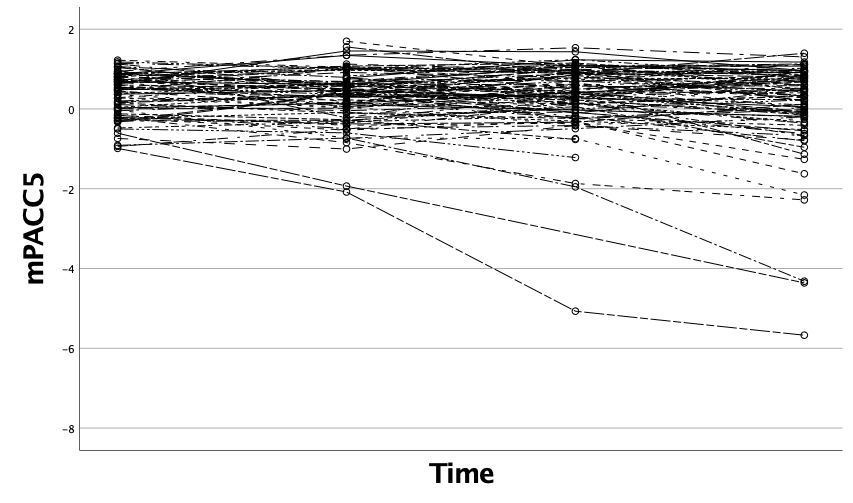


Supplementary Figure 1. Spaghetti plots showing individual trajectories over time for mean whole brain cortical thickness (A) and mPACC5 (B). For visualization purposes, we show 100 participants per plot.

Abbreviations: mPACC5 – modified Preclinical Alzheimer´s Cognitive Composite 5.

Supplementary Figure 2

 
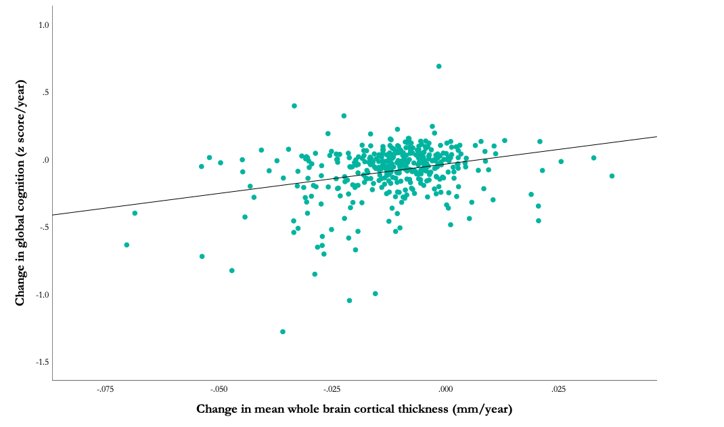


Supplementary Figure 2. Scatterplot showing the positive association between change in mean whole brain cortical thickness and change in global cognition.

Supplementary Figure 3

Supplementary Figure 3. Directed acyclic graphs (DAGs) constructed for inclusion of variables in mediation analyses.

Abbreviations: P-tau – phosphorylated tau; WML – white matter lesion; NfL – neurofilament light; Aβ – β-amyloid; GFAP – glial fibrillary acidic protein.
